# Improvement of the Insecticidal Capacity of Two *Purpureocillium Lilacinum* Strains against *Tribolium Confusum*

**DOI:** 10.3390/insects6010206

**Published:** 2015-03-18

**Authors:** Paula Barra, Miriam Etcheverry, Andrea Nesci

**Affiliations:** 1 Consejo Nacional de Investigaciones Científicas y Técnicas (CONICET), Puerto Madryn, 9120, Argentina; E-Mails: pbarra@exa.unrc.edu.ar (P.B.); metcheverry@exa.unrc.edu.ar (M.E.); 2 Laboratorio de Ecología Microbiana, Departamento de Microbiología e Inmunología, Facultad de Ciencias Exactas Físico Químicas y Naturales, Universidad Nacional de Río Cuarto, Río Cuarto, Córdoba, 5800, Argentina

**Keywords:** entomopathogenic fungi, insects, enzymes, virulence

## Abstract

Entomopathogenic fungi can regulate insect populations. They have extracellular enzymes that degrade cuticle components, mainly hydrocarbons, used as an energy source. The increase in insecticidal activity of fungi in a medium supplemented with cuticular hydrocarbons was assayed and the hydrolytic enzyme profiles of two strains of *Purpureocillium lilacinum* were evaluated. A spore suspension of *P. lilacinum* was inoculated in Petri plates with different values (0.99–0.97–0.95) of water activity (Aw) using the substrates gelatin, starch and tween-20. Growth rate on the different substrates and the enzymatic activity index for proteases, amylases and lipases at different incubation times, pH and Aw, was evaluated. Moreover, the insecticidal efficiency of strains grown in media supplemented with n-hexadecane and n-octacosane was analyzed. LT_50_ was calculated against adults of *Tribolium confusum* and showed that mortality increased about 15% when the strains grew in amended culture medium. High amylolytic activity was detected, but proteases were the main enzymes produced. Optimal protease production was observed in a range of acid and alkaline pH and lower Aw. The greatest growth rate was obtained in presence of gelatin. Lipase and amylase production was detected in small amounts. Fungal growth in media with hydrocarbon mixtures increased the pathogenicity of the two strains of *P. lilacinum*, with the strain JQ926223 being more virulent. The information obtained is important for achieving both an increase in insecticidal capacity and an understanding of physiological adaptation of the fungus.

## 1. Introduction

In Argentina about 6% of cereal production is lost at the post-harvest stage, due to flaws in the transportation and drying and mainly by insect damages [1,2]. Insect contamination in food commodities is an important quality control problem of concern for food industries [3].

Insects, such as *Tribolium confusum* (Jacquelin du Val) cause significant damage to stored maize [4]. Damage may be direct, such as weight loss, reduced germination and reduced nutritional value of grain or indirect such as heat and moisture migration, reservoir of disease and distribution of microorganisms [5]. Insects are involved in the colonization of grains, providing sites for fungal infection through the damage that can result in grains [6]. Also, previous studies have shown that certain insects that attack stored grains have the ability to disperse toxigenic *Aspergillus flavus* in those grains [7,8]. The interaction of these toxigenic fungi with substrate and abiotic predisposing factors may promote a moldy substrate and toxin accumulation in stored grains [9]. The main toxins produced by *A. flavus* are aflatoxins. The positive correlation between the consumption of food contaminated with aflatoxin B_1_ and increased incidence of liver cancer has led to the classification of this toxin as a carcinogen of the group 1 A by the International Agency for Research on Cancer [10]. For all these reasons, strategies to control insects are needed.

Currently, most of the management of post-harvest insect pests is carried out with synthetic chemical insecticides. However, the use of synthetic insecticides allowed in Argentine grains and in other countries is very limited [11]. It is known that these pesticides can adversely affect the environment. In addition, the continued use of these chemicals has resulted in serious problems such as resistance [12]. Hence, alternatives to conventional insecticides are needed. There are biologic agents with natural potential to protect crops. There is much interest in insect pathogenic fungi because they are considered to offer an environmentally friendly alternative to chemical pesticides [13,14,15]. Among these fungi, some belonging to the genus *Purpureocillium* have shown promising results for use as effective biocontrol agents [16,17,18,19]. In a previous study, all isolates of *Purpureocillium lilacinum* (Thom) Samson showed pathogenicity against insect vectors of aflatoxigenic *A. flavus* in stored maize [20].

Over the years, most of the studies for the development of a biocontrol agent have focused on the potential for inoculum production. Although these studies have been numerous, most of them have centered on optimizing the amount of propagules or mycelial fragments produced. However, little attention has been paid to the quality of the inoculum. Therefore, it is important to consider the existence of the real practical problem of the establishment and effectiveness of biocontrol agents in the natural environment. It is important that a biological control fungus has a tolerance for fluctuating abiotic factors like water activity and temperature [21].

All fungi need the physical presence of water for the diffusion of nutrients into the cell and the release of extracellular enzymes. However, water can be in the environment but not available. Under conditions of low water activity, fungi are able to accumulate polyols (sugar alcohols acyclic) at high concentrations. These polyols reduce the potential of intracellular water [22,23,24] producing a balance inside and outside the cell. Inhibition of enzymatic function by dehydration is also prevented [25]. Thus, water is available to maintain the turgor of the fungus. This allows the cell to continue operating efficiently. Therefore, physiological methods to improve tolerance to water stress are considered essential to enable the development of effective and consistent microbial biocontrol agents [26].

Early research has speculated that the mechanism of insect infection by fungi is through ingestion of the control agent [27]. However, over the years, it has been shown that entomopathogenic fungi infect the insect through the cuticle. For this reason, there is no need for these fungi to be ingested as they act as contact insecticides [28,29]. After binding the spore to the insect, the former germinate and penetrate the cuticle. This process occurs if factors, which influence the pathogenicity (water availability, temperature and nutrition) are favorable [30]. Also, it is necessary that a favorable environment is present for secretion of extracellular enzymes due to its hygroscopic nature [31]. Therefore, water availability is a critical factor for the germination of spores.

The entomopathogenic fungi produce a range of cuticle degrading enzymes corresponding to different polymers of the insect cuticle [32]. The insect cuticle is composed of a group of diverse constituents with variation in content and composition [33,34,35,36]. The cuticular composition has profound consequences on ecological and behavioral aspects of the insect. Hydrocarbons such as n-alkanes, alkenes and methyl-branched chains are common epicuticular lipids and have been widely studied [33,34,37]. Cuticular hydrocarbons can stimulate [38,39] or inhibit [40] the interaction between fungus and insect cuticle.

Fungal enzymes that hydrolyze proteins and chitin, which represent the main constituents of the insect cuticle, are considered vital to the infection process [32]. Many extracellular enzymes produced by fungi to degrade the outer cell layers of their hosts are regulated by carbon and nitrogen sources and pH [41]. The order of enzyme production is based on the constituents of the cuticle.

Starting from the biochemical mechanism of cuticular degradation and its potential as an alternative method of pest control, a hypothesis was proposed based on the probability that fungi with their mechanism of hydrocarbon assimilation enhanced could be directly related to increased virulence [42]. Therefore, hydrocarbons may induce germination besides functioning as catabolic substrates. Apart from this, the adaptation to different conditions like Aw, nutrients, pH and time could improve the pathogenicity and virulence of the fungus.

Therefore, the objective of this study was to stimulate the insecticidal efficacy of two *P. lilacinum* strains with proved efficacy against *T. confusum*. A first approximation of the enzyme profile of *P. lilacinum* was also evaluated.

## 2. Materials and Methods

### 2.1. Fungal Isolates

Two strains identified as *P. lilacinum* were used in these experiments. These strains were originally isolated from soil samples collected from the University of Río Cuarto Experimental Field Station in Río Cuarto, Córdoba, Argentina, and were identified and deposited in GenBank with the following accession number: JQ926212 and JQ926223. Both strains are pathogenic to *T. confusum*, an insect pest vector of aflatoxigenic fungi [20]. These strains are held in the Microbial Ecology Laboratory Collection, Microbiology and Immunology Department of the National University of Río Cuarto, Córdoba, Argentina.

### 2.2. Estimation of Enzymatic Activity of P. Lilacinum

#### Plate Assay Method

Protease, amylase and lipase activity was assayed by the plaque assay method [43]. The water activity (Aw) of the unmodified culture media was 0.99, and these were used as control treatments. The Aw was adjusted to 0.97 and 0.95 by addition of known amounts of non-ionic solute glycerol [44] and was determined using a water activity meter AquaLab (4TE model; AquaLab Technologies, Riverside, CA, USA). The activity of proteolytic and amylolytic enzymes of *P. lilacinum* was determined by using gelatin and starch, respectively, as substrate in a nutrient medium (0.5 g peptone, 0.3 g beef extract, 0.5 g NaCl, 1.5 g agar and 100 mL distilled water). For lipase activity, sorbitan monolaurate was added in nutrient medium (1 g peptone, 0.5 g NaCl, 0.1 g CaCl, 1.5 g agar and 100 mL distilled water) as a lipid substrate. Petri plates of different values of Aw and substrates were spot inoculated in the center of each plate, with *P. lilacinum* spores suspended in semi-solid agar [45]. Petri plates of the same values of Aw were sealed in polyethylene bags. The inoculated plates were incubated at 25 ± 1 °C for 15 days. At the end of the incubation period, the area of gelatin degradation was visible, flooding the plate with a saturated solution of mercury chloride reagent (15 g MgCl_2_ completely dissolved in 20 mL of 7 M HCl in 100 mL of distilled water). The mercury chloride solution reacts with the gelatin to produce a white precipitate that turns into a visible halo. To determine the starch degradation, the plates were flooded with iodine reagent (65 mg of iodine crystals and 130 mg of KI in 100 mL of distilled water). The lipolytic activity was evident by a precipitate, which was attributed to the production of fatty acids from lipids used as substrates by fungal enzymes. The diameter of each zone was recorded. Enzymatic activity (EA) was measured by the following formula [46]: EA = D−d; D = diameter of colony plus clearing precipitate zone; d = diameter of colony.

### 2.3. Growth Studies: Effect of Different Substrates on the Rate of P. Lilacinum Growth

The *P. lilacinum* strains grew, in each medium amended with different substrates for each enzyme, at 25 ± 1 °C during 15 days. The colony radius was measured daily during the incubation time. For each colony, two radii measured at right angles to one another, were averaged to find the mean radius for that colony. All colony radii were determined by using three replicates for each one. The radial growth rate (mm d^−1^) was subsequently calculated by linear regression of the linear phase for growth in relation to the carbon source of the culture medium and the water activity.

### 2.4. Quantitative Studies for Estimating the Production of Extracellular Enzymes by P. Lilacinum

#### 2.4.1. Culture Media

Both strains of *P. lilacinum* were cultured in two minimal growth media, medium 1 (0.4 g yeast extract, 0.6 g K_2_HPO_4_, 0.6 g MgSO_4_, 0.6 g NaCl and 100 mL distilled water) and medium 2 (0.11 g FeSO_4_, 0.04 g ZnSO_4_, 0.05 g MgSO_4_, 0.2 g KNO_3_ and 100 mL distilled water). Medium 1 was used to stimulate the production of proteases and amylases, while lipases were produced in Medium 2. Each culture medium was supplemented with 1% gelatin, starch or tween-20, depending on the enzyme, which was to be evaluated. The Aw of the unmodified media was 0.99, and these were used as the control treatments. The Aw was adjusted to 0.97 and 0.95 as described above. The media were adjusted to pH 3, 7, 9 and 11 adding the necessary quantity of the following buffer solutions: citric acid 0.1 M + sodium hydroxide 1 M (5:1) (sol A)/hydrochloric acid 0.1 M (sol B); potassium monohydrogen phosphate 0.2 M (sol A)/potassium dihydrogen phosphate 0.2 M (sol B); glycine 1 M (sol A)/sodium hydroxide 1 N (sol B). Appropriate amounts of solutions A and B were used. To achieve pH 3, 40 mL sol A + 60 mL sol B of the first buffer solution were mixed. To achieve pH 7, 39 mL sol A + 61 mL sol B of the second buffer solution were used and to achieve pH 9 and 11, 85.03 mL sol A + 14.96 mL sol B and 52.3 mL sol A + 47.7 mL sol B of the third buffer solution were used, respectively.

#### 2.4.2. Experimental Conditions and Fungal Inoculation

Hundred milliliters of each culture medium with different levels of Aw and pH were placed into flasks of 250 mL to estimate the production of each enzyme. Each flask was inoculated with 1 mL of spore suspension of *P. lilacinum* (10^7^ spores mL^−1^). The inoculated culture media were incubated on a rotary shaker (150 rpm) at 25 ± 1 °C. After 3, 7, 10 and 15 days enzyme production was measured as follows:

##### 2.4.2.1. Spectrophotometric Analysis of Proteases and Amylases

Total protein concentration was measured according to biuret protein assay [47]. A standard curve was calculated using bovine serum albumin (BSA) in a concentration range from 0.25 to 1 mg mL^−1^. Standard solution was prepared at a concentration of 1 mg mL^−1^. Absorbance was measured in a spectrophotometer at 545 nm. Aseptically collected aliquots from each flask were centrifuged at 13,000 rpm for 10 min. The supernatant was used as crude enzyme extract (CEE). It was mixed with 1 mL of biuret reagent (3.8 g CuSO_4_-5H_2_O, 6.7 g EDTA, 200 mL NaOH 5N, 1 g KI, and 700 mL distilled water) and vortexed. After 5 min, the absorbance was determined. One mL of buffer without inoculation was used as control. During the determination of protein in each sampling time, samples were kept at 4 °C to preserve the function of the enzyme.

The amylolytic enzyme concentration was determined according to Miller [48] with some modifications. Aseptically collected aliquots from each flask were centrifuged at 5000 rpm for 5 min. The supernatant was used as crude enzyme extract (CEE). Appropriately diluted enzyme was mixed with equal amounts (1mL) of starch solution (1 g starch and 100 mL distilled water). The reaction was allowed to proceed by incubating at 27 ± 1 °C for 15 min. At the end of the incubation period, the reaction was stopped by addition of 6 mL of water. Enzyme activity was detected as absorbance at 540 nm. Control tubes were prepared by addition of sterile distilled water instead of CEE.

##### 2.4.2.2. Tritimetric Method

Lipase concentration was determined using a tritimetric assay [49] with NaOH 0.05 M, using emulsified oil as substrate. One milliliter of appropriately diluted enzyme was added to 5 mL of emulsion containing 25% (v/v) olive oil, 75% (v/v) arabic gum and 2 mL of 10 mM phosphate buffer solution at pH 7. Phenolphthalein in alcohol + water (1:1) was used as indicator. The assay was performed at 37 ± 1 °C for 30 min. The reaction was stopped by addition of 15 mL of acetone/ethanol (1:1 v/v). The amount of fatty acid released due to the enzyme activity was titrated against NaOH 0.05 M until the color of the reaction mixture changed to pink. A lipase unit was defined as the enzyme able of releasing 1 mol of fatty acid at 37 ± 1 °C and pH 7, per minute. One mL of titration volume is equal to 2.5 units of lipase.

### 2.5. Stimulation of Pathogenicity against T. Confusum

#### 2.5.1. Fungal Growth on Synthetic Hydrocarbon-Enriched Media

The control culture medium (CM) of this study contained 0.4 g KH_2_PO_4_, 1.4 g NaHPO_4_, 0.6 g MgSO_4_, 1 g KCl, 1.4 g NH_4_NO_3_, 20 g glucose, 10 g yeast extract, 15 g agar and 1000 mL distilled water. The amended medium (SM) had a similar composition to the CM medium but glucose and yeast extract were replaced by 20 mL of hexane solution composed of 15% w/v of n-hexadecane and/or n-octacosane (Sigma Aldrich, St. Louis, MO, USA) [50]. The Aw of the unmodified media was 0.99, and these unmodified media were used as the control treatments. The Aw was adjusted to 0.97 and 0.95 as described above. A spore suspension (10^7^ spores mL^−1^) of each entomopathogenic fungus strain was inoculated onto Petri plates containing CM and SM culture media with the purpose of stimulating their virulence. The Petri plates of the same Aw values were sealed in polyethylene bags and incubated at 25 ± 1 °C during 15 days.

#### 2.5.2. Insect Rearing

Cultures of one strain of the confused flour beetle *T. confusum* (Jacquelin du Val) were obtained from the Department of Agricultural Zoology, Faculty of Agronomy, University of Buenos Aires, Argentina. Mixed-sex adults 1–3 weeks of age were used in the test. Insects were reared on a diet of wheat flour, cornstarch and yeast (10:10:1.5) in plastic containers with 200 g of the mixture. Insects were reared at 27 ± 1 °C, 70 ± 5% relative humidity and photoperiod of 12:12 h light:dark cycle.

#### 2.5.3. Pathogenicity Bioassay

Twenty *T. confusum* adults were treated by immersion for 30 s in 600 µL of a spore suspension (10^7^ spores mL^−1^) [51] of either of the two *P. lilacinum* fungal strains that were cultured using both CM and SM media. After treatment, beetles were placed in a plastic jar with 500 g of maize grains [52]. Jars were placed in a chamber under controlled conditions at 27 ± 1 °C, 70 ± 5% r.h, with a photoperiod of 12:12 h light:dark cycle [53]. Mortality was recorded daily for 20 days and analyzed by probit analysis to obtain lethal time (LT_50_) values for comparison with controls. Fungal mortality was confirmed by placing dead beetles on water agar (15 g l^−1^ agar and 1000 mL distilled water) and incubated at 25 ± 1 °C for 7 days.

### 2.6. Statistical Analysis

The analysis of variance in a completely randomized design was used to compare the effect of fungal strain, substrate, water activity, incubation time and pH on enzymes production, and the effect of the nutritional modification on insecticide efficiency of *P. lilacinum* against *T. confusum*. The statistical program, InfoStat, for Windows version 2008 (Group InfoStat, FCA, National University of Córdoba, Argentina) was used [54].

## 3. Results

### 3.1. Extracellular Enzymes Activity by P. Lilacinum

**Figure 1:**
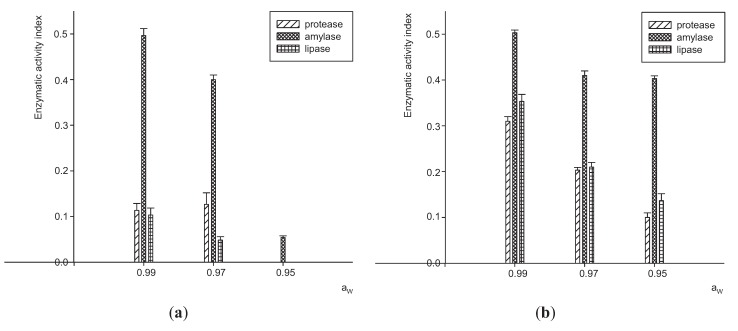
Extracellular enzyme activity index of two strains of *P. lilacinum* (**a**) JQ926212 and (**b**) JQ926223. Tuckey test (*P* < 0.05) was used to compare different enzymatic activity for the same Aw.

An analysis of the influence of substrate in enzymatic activity was performed. The enzymes values produced varied by Aw and strain (*P* < 0.0001). Amylolytic activity was observed with both fungal strains at 3 Aw tested. The highest yield was obtained at 0.99 Aw. This activity was significantly greater than the other activities analyzed (*P* < 0.05). In general, all values of enzymatic activities were lower when Aw decreased. No protease and lipase activity for the JQ926212 strain at 0.95 Aw was observed. It was observed that the JQ926223 strain had higher activity than the JQ926212 strain for all enzymes assayed (Figure 1).

### 3.2. Effects of Aw and Substrates on Growth

**Figure 2:**
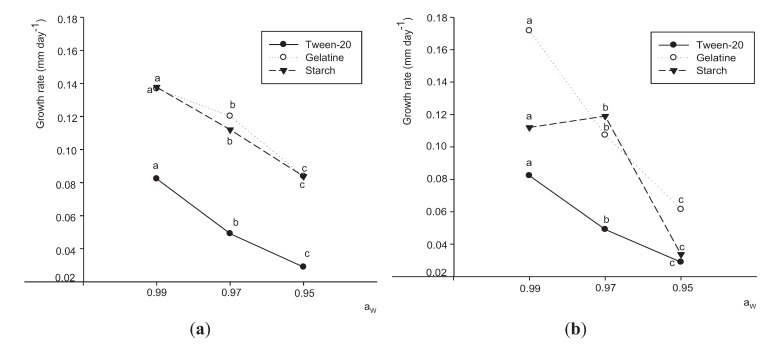
Comparison of growth rates of *P. lilacinum* JQ926212 (**a**) and JQ926223 (**b**) with different substrates: ●: tween-20; ○: gelatine and ▼: starch at different water activities. Data with different letters for the same substrate at different Aw are significantly different based on the Tukey test (*P* < 0.05).

Changes in growth rate of both strains in relation to Aw level and different substrates at 25 °C are shown in Figure 2. The major effect was that of Aw (F = 120.17; *P* < 0.0001). The growth rate of both strains was inhibited with decreasing Aw. Apart from this, both strains showed lower growth rates in the culture medium supplemented with tween-20. The strain JQ926223 showed a greater growth rate in presence of gelatine as substrate at the highest and lowest Aw tested. However, at 0.97 Aw, the growth rate was greater in presence of starch. The opposite was observed for strain JQ926212 in presence of gelatine and starch. It was observed that strain JQ926223 was more sensitive to changes of Aw for all substrates. A reduction of about 40% was observed when the fungus grew on the medium with gelatine in the Aw range of 0.99–0.97 and 0.97–0.95 Aw.

### 3.3. Extracellular Enzyme Production by P. Lilacinum

**Figure 3:**
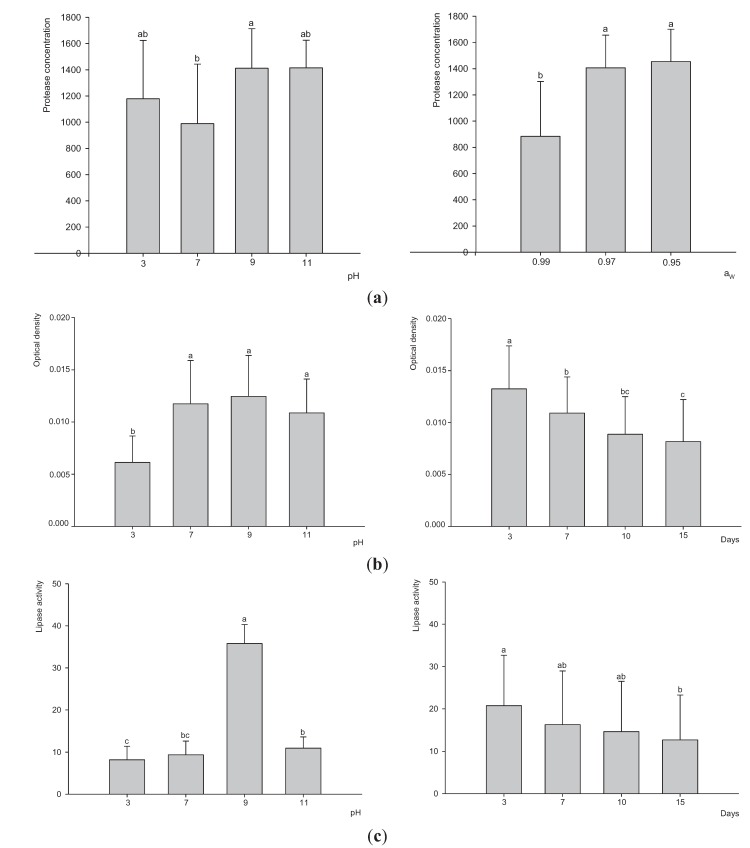
Production of proteases (**a**), amylases (**b**) and lipases (**c**) by both strains of *P. lilacinum* JQ926223 and JQ926212. Bars represent means and standard errors for each condition of Aw, pH and incubation time (days). Data with different letters for each Aw, pH or incubation time are significantly different based on LSD Fisher test (*P* < 0.05).

The determination of the optimal conditions for the production of higher yields of extracellular enzymes was carried out by analyzing the influence of factors, such as pH, Aw and incubation time. No significant differences between strains (F = 0.05; *P* = 0.8301) were observed; thus Figure 3 summarizes the effect of different factors on the two fungal strains. Moreover, no effect was observed for incubation time for protease activity or for Aw for amylase and lipase activities. Therefore, the effect of these two factors is not shown. The maximum protease concentration was achieved at alkaline and acidic pH and in the range of 0.97–0.95 Aw (Figure 3a). The pH (F = 23.29; *P* < 0.0001) and incubation time (F = 14.88; *P* < 0.0001) were the two factors that affected the amylolytic production the most. The optimum pH range for the production of amylases was alkaline. A good yield at neutral pH was also obtained. The maximum amylase production was obtained at the shortest incubation time (Figure 3b). Lipase production was affected by incubation time (F = 106.1; *P* < 0.0001) and pH (F = 343.1; *P* < 0.0001). The greatest amount of these enzymes was obtained after 3 days of incubation and pH 9 (Figure 3c).

### 3.4. Pathogenicity Analysis

**Figure 4:**
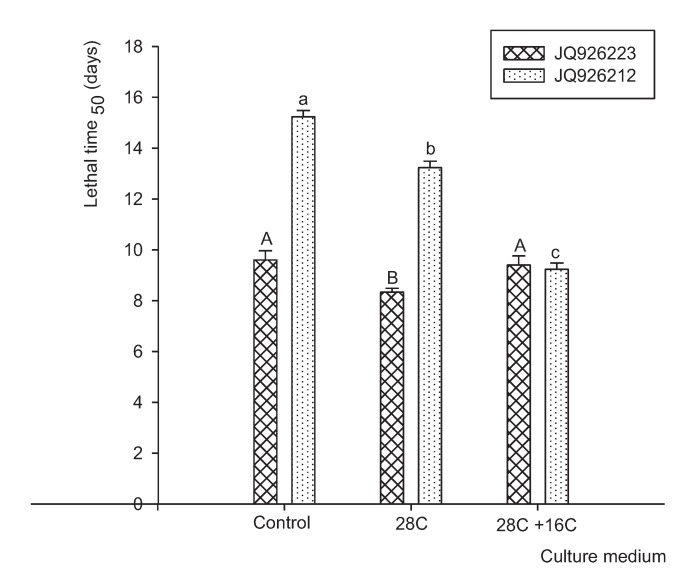
LT_50_ for *T. confusum* adults exposed during 15 days to two strains of *P. lilacinum* JQ926212 and JQ926223 grown in control medium and amended medium with 28C and 28C + 16C mixture. Data with different letters for each strain (JQ926223: capital letters, JQ926212: small letters) at different treatments are significantly different based on the Tukey test (*P* < 0.05).

Statistical analyses of the effects of *P. lilacinum* strains, growth media and their interaction on insecticide efficiency against *T. confusum* showed significant differences (*P* < 0.0001). The major effect was that of strains (F = 242.08). Figure 4 shows the LT_50_ of both strains in the control medium and the medium amended with hydrocarbons. The LT_50_ for both strains of *P. lilacinum* after growth in the presence of C28 and C28 + C16 mixture was 10.9 and 9.6 days, respectively, while LT_50_ in the control medium was 12.5 days. The mortality in control treatments was low (<10%), and no mycosis was detected on any insects. Strains JQ926223 and JQ926212 showed a mortality rate of 70% and 50%, respectively, when these strains were grown in control medium. Furthermore, the mortality rate increased when both strains were grown in amended medium, showing a mortality rate of 80% and 70% for JQ926223 and JQ926212, respectively.

## 4. Discussion

The assessment of an organism for microbial control in a pest management strategy requires basic studies such as isolation, culturing, biological testing and prediction of effects on the pest population and the environment [55].

Virulence of entomopathogenic fungi is often related to adhesion, rate of germination and growth of conidia on the cuticle of the insect hosts. This aspect reflects the metabolic advantages of degrading the cuticles of insects that can be seen in fungi stimulated by subculture in culture media amended with hydrocarbons [56,57,58]. This stimulation gives an initial advantage during the infective process. Among a variety of factors involved in the specificity of the interaction between the cuticles of insects and entomopathogenic fungi, the composition of the components of the host cuticle could stimulate or inhibit fungal growth [50]. Therefore, an increase of host mortality by inducing the synthesis of fungal degrading enzymes, suggests a possible approach for mycoinsecticide improvement. In our study, JQ926223 is more virulent than JQ926212 and both are pathogenic to *T. confusum*. However, mortality rate varied according to the culture medium used for fungal development. About 15% of increase in mortality was obtained when *P. lilacinum* strains grew in culture media supplemented with hydrocarbons.

Pedrini *et al.* [59] confirmed the results of Crespo *et al.* [50] regarding the increase of susceptibility of *Acanthoscelides obtectus* (60%–100% of mortality) to *Beauveria bassiana* after developing on media amended with hydrocarbons. A significant increase in mortality, reaching 90%, was also detected in *Rhyzopertha dominica*, therefore this method may increase the virulence favoring the early stages of the infection [50,60,61]. This increase is evidenced both by an increase in the percentage of mortality [50] as well as by a decrease in lethal time [61].

After the adhesion and germination of conidia occurred, penetration through the cuticle is the next stage of the infection cycle. The degradation of the hydrocarbons begins with the reaction of enzyme complexes. Information of fungal enzymatic activities involved in insect penetration would be required for the development of efficient mycotic agents for insect biological control [62]. Accordingly, in this study, we have analyzed the relationship of enzymatic activities with the fungus pathogenicity against *T. confusum*. Extracellular enzymes that degrade compounds in the cuticle of insects [63], such as proteases, chitinases and lipases are in some way related to the pathogenicity of entomopathogenic fungi [64]. However, other studies showed no correlation between enzyme activities and fungal pathogenicity [65].

In our study, high amylolytic activity was present in both strains. Protease and lipase activities were also present in lesser amounts. According to Esteves *et al.* [66], lipolytic activity of entomopathogenic fungi is low, although they suggest that lipases might have been synthesized later in time. They also conclude that lipase enzymes are secreted in little amounts when compared with the production of other extracellular enzymes. Moreover, the relatively low protease activity obtained in our study could be due to the fact that gelatin induced the production of chitinases and estearases, but surprisingly did not increase the production of proteases. The source of the substrate would favor the production of other enzymes than the proteases [66]. The use of high concentrations of gelatin repressed protease activity, such as albumin, whereas fibrous collagen enhanced protease activity [67].

It is known that enzymatic activity is directly related to fungal growth, which shows that the mycelial growth is crucial for an adequate production of different extracellular enzymes [68]. To determine the optimal conditions of physiological adaptation of the entomopathogenic fungus, it is important to find the best substrate to achieve the highest growth.

It is also important to compare if these optimum conditions match the increasing enzyme production. In this study, the greater growth rate was in presence of gelatin as substrate at the higher and lower Aw tested. On the other hand, both strains showed lower growth rates in the culture medium supplemented with tween-20 tested at 3 Aw. Both strains showed an acceptable growth rate with starch, probably because these strains are adapted to remain in the soil during the stages that not parasitize insects [69].

In this study, it was observed that the amount and type of enzymes secreted by *P. lilacinum* were different depending on the pH and Aw of the culture medium and the time of incubation. Therefore, cultural conditions appear to have an important effect on the results obtained.

Proteases secreted during the early stages of penetration [70] are considered the most important pathogenicity factor, because proteins are the major component in the exoskeleton (61%–70%) [71,72] and the cover of the eggs [73,74]. In our study, proteases were the main enzymes produced by these fungi. The mean values obtained were between 800 and 1400 mg/mL that were kept constant during the incubation time. The higher production of proteases was obtained in the range of acid and alkaline pH and lower Aw evaluated (0.97–0.95). The incubation time showed no statistically significant effect on the production. These results do not agree with those reported by Ghanem *et al.* [75] who showed that the highest protease production was recorded after 96 h of growth. But the production was not kept constant. Gomez Fernandez *et al.* [76] showed that *Metarhizium anisopliae*, *B. bassiana* and *Paecilomyces sp*. were good producers of proteases. Such production was enhanced when fungi were cultured in the absence of glucose, with gelatine as the only carbon source. Petlamul and Prasertsan [77] showed that *M. anisopliae* and *B. bassiana* had high protease activity during the first 4.5 to 6 days of incubation. While incubation time elapses, the protease activity decreases, probably for nutrient limitation or cell autolysis [78].

Different studies have shown that each entomopathogenic genus has a different enzymatic apparatus, with *Paecilomyces sp*. showing the greatest versatility for different enzymatic substrates. Other studies involving strains of the genus *Pochonia* have shown that this genus is also a producer of extracellular proteases in the presence of gelatine [79,80,81,82].

Macheronni Jr. *et al.* [83] showed production of lipase at neutral and alkaline pH, but no enzyme production was detected at acidic pH. Similar results were reported by Silva *et al.* [84], when they evaluated the production of lipase by *M. anisopliae* in presence of various carbonaceous sources. These authors suggest a relationship between these enzymes and the pathogenicity of the fungus. In our study, lipase production was low; the maximum lipolytic production was recorded after 3 days of incubation and alkaline pH (36 U/mL). Hasan *et al.* [85] showed that the maximum lipase production of *Verticillium lecanii* was found at pH 7 (55 U/mL). Results obtained by other authors and the results of this study are consistent with St. Leger *et al.* [86] who observed large variations in levels of enzymatic production among different entomopathogenic fungi.

In our study, the amylases were also detected but in small quantities. This could be due to a lack of production of enzymes or an intracellular location, because strains grew well in culture media supplemented with starch. The optimal amylolytic production was at 3 days of incubation and pH 9; however similar results were found at neutral pH. These results do not coincide with what Hasan *et al.* [85] observed. They reported the highest amylolytic production in strains of *V. lecanii* on the seventh day of incubation at pH 3. Lopez-Llorca and Carbonell [87] obtained similar results in the production of extracellular amylase by strains of *V. lecanii* isolated from soil and insect samples. According to Jackson *et al.* [64] there is an inverse relationship between the amylolytic activity and pathogenicity. There are at least two hypotheses for these results: (a) an absence of amylase production may be fortuitously associated with some virulence factor of the fungus (b) non-virulent isolates are predominantly saprophytes and are likely to require high amylolytic activity to achieve degradation of organic debris in soil.

## 5. Conclusions

The fungal growth with hydrocarbon mixtures increased the pathogenicity of the two strains of *P. lilacinum*, with the strain JQ926223 being more virulent. Both strains displayed mainly protease activity. The strains showed acceptable protease production in a range of pH values from acidic to alkaline and lower Aws.
